# Deep Learning-Based Algorithm for Automatic Detection of Pulmonary Embolism in Chest CT Angiograms

**DOI:** 10.3390/diagnostics13071324

**Published:** 2023-04-03

**Authors:** Philippe A. Grenier, Angela Ayobi, Sarah Quenet, Maxime Tassy, Michael Marx, Daniel S. Chow, Brent D. Weinberg, Peter D. Chang, Yasmina Chaibi

**Affiliations:** 1 Department of Clinical Research and Innovation, Foch Hospital Suresnes, Versailles Saint Quentin University, 78000 Versailles, France; 2 Avicenna.AI, 13600 La Ciotat, France; 3 Department of Radiological Sciences, University of California Irvine, Irvine, CA 92697, USA; 4 Center for Artificial Intelligence in Diagnostic Medicine, University of California Irvine, Irvine, CA 92697, USA; 5 Department of Radiology and Imaging Sciences, Emory University, Atlanta, GA 30322, USA

**Keywords:** pulmonary embolism, computed tomography angiography, artificial intelligence, deep learning tool, chest CT

## Abstract

Purpose: Since the prompt recognition of acute pulmonary embolism (PE) and the immediate initiation of treatment can significantly reduce the risk of death, we developed a deep learning (DL)-based application aimed to automatically detect PEs on chest computed tomography angiograms (CTAs) and alert radiologists for an urgent interpretation. Convolutional neural networks (CNNs) were used to design the application. The associated algorithm used a hybrid 3D/2D UNet topology. The training phase was performed on datasets adequately distributed in terms of vendors, patient age, slice thickness, and kVp. The objective of this study was to validate the performance of the algorithm in detecting suspected PEs on CTAs. Methods: The validation dataset included 387 anonymized real-world chest CTAs from multiple clinical sites (228 U.S. cities). The data were acquired on 41 different scanner models from five different scanner makers. The ground truth (presence or absence of PE on CTA images) was established by three independent U.S. board-certified radiologists. Results: The algorithm correctly identified 170 of 186 exams positive for PE (sensitivity 91.4% [95% CI: 86.4–95.0%]) and 184 of 201 exams negative for PE (specificity 91.5% [95% CI: 86.8–95.0%]), leading to an accuracy of 91.5%. False negative cases were either chronic PEs or PEs at the limit of subsegmental arteries and close to partial volume effect artifacts. Most of the false positive findings were due to contrast agent-related fluid artifacts, pulmonary veins, and lymph nodes. Conclusions: The DL-based algorithm has a high degree of diagnostic accuracy with balanced sensitivity and specificity for the detection of PE on CTAs.

## 1. Introduction

Estimated at around 180,000 deaths per year, pulmonary embolism (PE) remains a leading cause of death in the United States [1]. It is the third most common cause of cardiovascular disease events, with only myocardial infarction and stoke having a higher prevalence [2]. Prompt recognition of acute PE and immediate initiation of anticoagulation and mechanical thrombectomy can greatly reduce the risk of death [3]. The most common cause of death from PE is a failure of diagnosis [4]. The clinical presentation of PE is often nonspecific and can be mimicked by a range of other conditions. The diagnostic approach to PE usually involves a series of investigations including echocardiography and D-dimer, establishing the necessity for further confirmatory examinations like chest CT angiography (CTA) and ventilation/perfusion scans [5]. CTA has become the gold standard diagnostic modality for PE and has almost eliminated the use of ventilation/perfusion scans [6]. CTA is a non-invasive, widely available, and rapidly performed modality. However, the diagnosis of PE in CTA is time-consuming and requires the expertise of radiologists. As a result, the interpretation process is susceptible to errors and delayed diagnosis [7]. The usage of CTA in emerging departments has increased over 27-fold in the last two decades [8,9]. Variations in concordance between general and subspecialist radiologists have been reported as well as between residents, fellows, and attending radiologists [10]. The workload increases in emergency settings, and fatigue from long work hours and overnight shifts may contribute to an increase in diagnostic errors [11,12,13]. Studies have shown that there is up to a 13% discrepancy rate between overnight radiologists and the day faculty [14,15,16]. The radiologist’s sensitivity for detecting PE has been measured in the range of 67 to 87% with a specificity of 89 to 99% [17,18,19]. Studies have reported promising results in applying deep learning (DL) models to automate PE diagnosis on CTA [20,21,22,23,24,25]. In a systematic review with the meta-analysis of DL-based AI algorithms developed to detect PE on CTA, published in 2021, five studies provided enough data to calculate the test accuracies [26]. A pooled sensitivity of 88% per scan and a specificity of 86% per scan were shown. Furthermore, the authors noticed a patient selection bias in more than half of the studies with a failure of the study population description. The question on the potential generalizability of such algorithms remains on hold. Actually, generalization across institutions, particularly in the setting of varying CT scanner models and reconstruction methods, present another difficulty in the generalization of automated diagnosis.

The objective of our study was to validate a commercially available AI-powered application, named CINA-PE v1.0.3, for suspected PE triage and prioritization. The algorithm was trained and tested on data from multiple clinical sites and vendors. The validation of the application presented in this study was performed on CTA exams acquired on various CT scanners from multiple clinical sites through the U.S. The diagnostic performance of the device to detect PE was measured by a comparison with the ground truth established by several expert U.S. board-certified radiologists. Furthermore, an evaluation of the triage effectiveness was performed by measuring the standalone per-case processing time (time-to-notifications) of the chest CTA images. Our results demonstrated good performance of this AI-based algorithm, opening the way for its use to help radiologists by highlighting the exams that are positive for PE in the worklist, thereby accelerating the diagnosis and communication workflow.

## 2. Material and Methods

### 2.1. AI Algorithm: General Architecture, Training, and Testing

Convolutional neural networks (CNNs) were used to design the application. The algorithm used a hybrid 3D/2D UNet [21,22] topology comprising four stages. The first stage extracted the lung and main pulmonary artery pixels from the pixel volume. Then, the region proposal network generated a first segmentation of PEs, which were then sharpened by the false positive network using a CNN region proposal network. The post-processing stage eliminated false-positives outside the lung and the main pulmonary artery, and finally, a logic function between all voxel-wise masks was used to establish the final decision.

For the training phase, a K-fold cross validation schema was implemented, allowing us to pre-compute masks for each fold without introducing bias. A dataset of 150 CTAs with a balanced proportion of healthy lungs and lung diseases (pneumonia, ground-glass opacity, pleural effusion, etc.) was used to train the lung and main pulmonary artery segmentation algorithm. Then, a dataset of 4795 CTAs was used to train the region proposal network and false positive reduction networks. A masked softmax cross entropy was used as the loss function. The datasets were provided from several U.S. clinical centers and were adequately distributed in terms of vendors (Canon Medical Systems Corporation, GE Healthcare, Philips Healthcare, Siemens Healthineers), patient age, slice thickness, and kVp. The training datasets were provided by an American Teleradiology Company on behalf of a data sharing partnership. The ground truth of the training phase was established by an expert on a per-slice segmentation level.

The testing phase was performed using 109 (62 positive) CTAs adequately distributed in terms of vendors, patient age, slice thickness, etc. A total of 2 FP and 2 FN were observed, leading to an overall sensitivity of 96.8% [88.8–99.6%], specificity of 95.7% [85.5–99.5%], and accuracy of 96.3%.

### 2.2. Data Selection for Validation

This is a retrospective multicenter and blinded standalone performance study. All validation anonymized data were received from the same data sharing partnership with the American Teleradiology Company from 228 U.S. clinical sites. The validation dataset was different from the one used for the algorithm’s training and testing, and have never been used in any way in the development of a software device. A waiver of consent was obtained from the Western Institutional Review Board for all cases. Informed consent for participation was not required for this study in accordance with the national legislation and institutional requirements. 

The PASS sample size (Kaysville, UT, USA) software was used to calculate the minimum number of cases needed to achieve a 95% CI lower bound of at least 80%, assuming a point estimate of 90% (for sensitivity and specificity, separately). Using the binomial dichotomous endpoint for one sample study, at least 137 positive and 137 negative anonymized cases were required.

The Teleradiology company used a specific tool (Natural Language Processing of radiology reports) to find relevant examinations (i.e., suspected positive PE and potentially negative ones) to send approximately the same portions of positive and negative cases. Among the received data, we consecutively preselected the cases that were compatible with the recommended requirements summarized in the list of inclusion criteria for the PE study (Table 1). A total of 387 anonymized CTA cases were considered for the standalone performance validation of the automated device.

### 2.3. The Ground Truth

Two U.S. board certified expert radiologists (DC and BW), blinded to each other, proceeded with the visual analysis of the selected chest CTA datasets to determine whether there was (or not) a suspected PE on the CTAs. Positive cases were considered when any acute or chronic occlusion (marginal or endoluminal filling defect) was seen within the main, lobar, interlobar, and/or segmental arteries only, since the occlusions of the subsegmental arteries were not targeted by the software. Consequently, if the operators noticed only the presence of occlusions of the subsegmental arteries, they were asked to label the case as being negative. In the case of discrepancy between both radiologists, a third U.S. board-certified radiologist (PC) reviewed the axial CTA series. The final ground truth (presence or absence of PE) was established by majority consensus. Additionally, the operators were asked to report any confounding condition (i.e., streak artifact, motion artifacts, presence of tumor, pleural effusion, catheter etc.) if observed.

### 2.4. Post-Processing

The next step consisted of processing the same selected anonymized dataset by a U.S. Food and Drug Administration (FDA) cleared and CE-marked AI-powered application for suspected PE triage and prioritization, named CINA-PE (v1.0.3, Avicenna.AI, La Ciotat, France), which is the subject of the current standalone performance validation study. All received data were automatically processed by the application, and notifications of the suspected findings (if any) were automatically displayed along with the image series information. No visualization of the data, or any interpretation of the displayed images was required for this step analysis. The results were automatically computed and collected for analysis. The evaluations were performed blind, without access to the results of the U.S. board-certified radiologists’. For all CTA cases, the notification time, time between the end of the DICOM reception (made available for PE image processing), and the end of processing (positive or negative identification) were measured.

### 2.5. Statistical Analysis

The comparison between the results provided by the U.S. board-certified radiologists and those automatically computed by CINA-PE was performed according to the numbers of the true positive (TP), true negative (TN), false positive (FP) and false negative (FN) values. Sensitivity, specificity, accuracy, and area under the receiver operating characteristic curve (ROC AUC) were computed for the entire cohort. The 95% confidence intervals (95% CI) were calculated for sensitivity and specificity using the exact binomial distribution (Clopper–Pearson). The lower bound of the two-sided 95% CI were compared to a performance goal of 80% for sensitivity and specificity. Furthermore, the Matthews correlation coefficient (MCC) was calculated to measure the quality of binary classifications, which generates a high score only if the binary predictor is able to correctly predict the majority of positive and negative data instances, and returns a value between −1 and +1, where a coefficient of +1 represents a perfect prediction.

Positive and negative predictive values were also computed using sensitivity and specificity with varying prevalence values (from 10% to 50%, increment of 5%).

A stratified statistical analysis on the imaging acquisition parameters (scanner makers, number of detector rows and slice thickness) and the patient groups (age, sex, and U.S regions) was also calculated (Supplementary Materials—Table S1).

Additionally, PE prioritization and triage effectiveness (mean ± SD, lower and upper 95% CI limits, median, minimum, and maximum values) were evaluated by the standalone per-case processing time of the device.

## 3. Results

### 3.1. Data Distribution

The mean ± SD age of the 387 patients included in the performance study was 62.5 ± 16.3 y/o (min = 19 y/o and max = 90 y/o). Male and female populations were almost equally distributed (53% and 47%, respectively). The clinical data were acquired in 228 cities, located in 43 different states. For stratified analysis, the 387 cases were grouped by U.S. regions (Pacific, Continental, Northeast, and Southeast). The CTA examinations were performed on 41 different scanners including 17, five, 14, four, and one different models from GE Healthcare, Philips Healthcare, Siemens Healthinners, Canon Medical Systems Corporation (formerly Toshiba), and Philips-Neusoft Medical Systems (PNMS), respectively. The number of detector rows varied from 8 to 320. For 25 (6.5%) cases, the number of detector rows was not available. All data (100%) had slice thickness values lower or equal to 2.5 mm, as recommended for an optimal use of the PE triage application.

### 3.2. Ground Truth Results

Among the 387 included CTA cases, one U.S. board-certified radiologist (BW) assessed 192 cases as being positive and 195 as being negative, whereas the second U.S. board-certified radiologist (DC) annotated 188 cases as being positive and 199 as being negative for the same cohort. Disagreements were observed between both operators for 30 (7.8%) cases (very good inter-rater agreement was computed according to Cohen’s Kappa = 0.84 [95% CI: 0.79–0.89]). After a review of the discrepant cases by the third radiologist (PC), the ground truth established by majority consensus was 186 (48.1%) positive and 201 (51.9%) negative cases. The distribution of the PE cases in terms of positives and negatives for different categories (i.e., scanner makers, patient age and sex, U.S. regions, etc.) are presented in Supplementary Materials—Table S1.

### 3.3. Sensitivity, Specificity, AUC, Accuracy, and MCC

**Figure 1:**
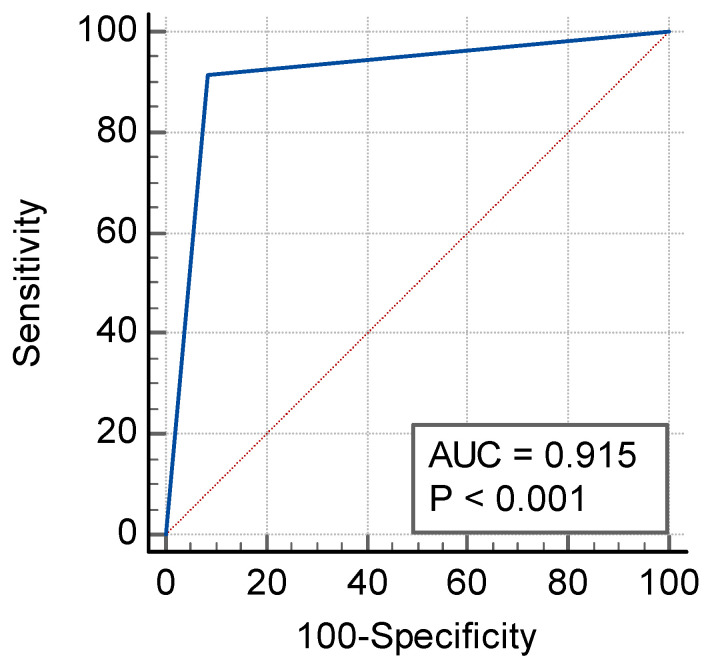
Receiver-operating characteristic (ROC) curve for CINA-PE.

**Figure 2:**
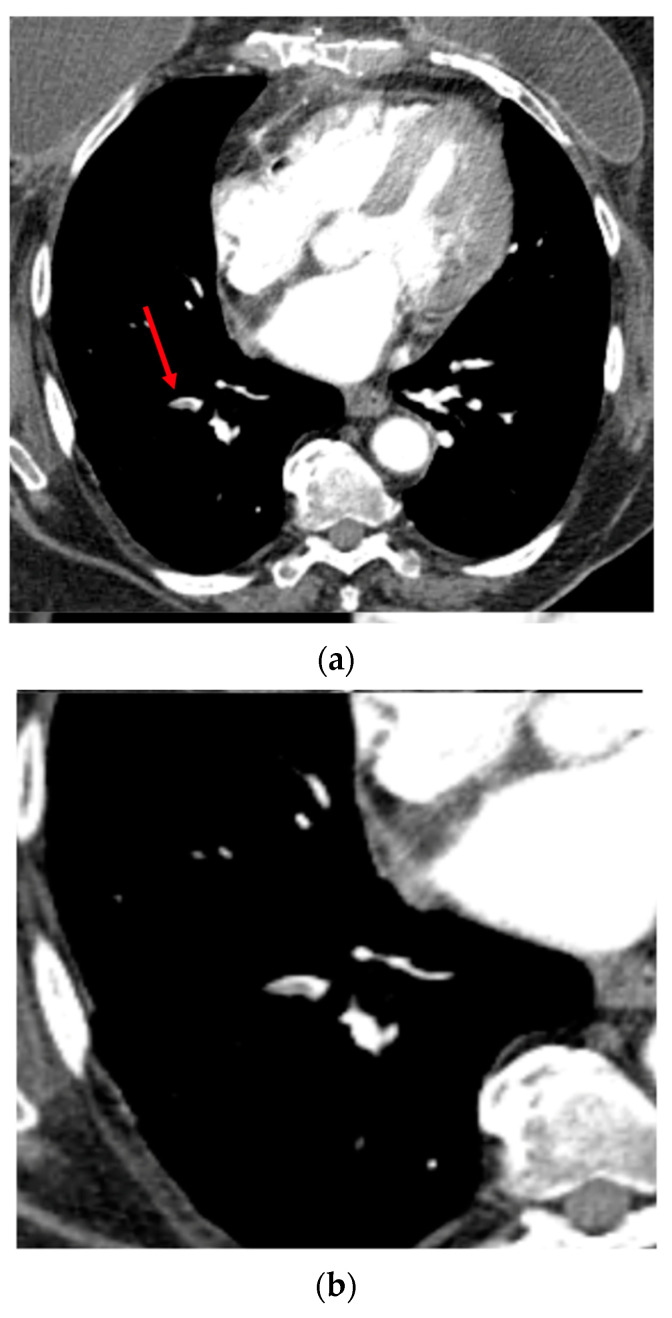
Axial CTA image. Example of segmental pulmonary embolus (PE) in the right lower lobe (filling defect within the arterial lumen), correctly detected by the CINA-PE software considered as a true positive case. Top (**a**): The red arrow shows the clot within the arterial lumen. Bottom (**b**): Axial image targeted on the right lower lobe.

The AI-powered algorithm achieved its primary endpoint of lower 95% CI bounds ≥80% for sensitivity and specificity. As presented in Table 2, the sensitivity was 91.4% [95% CI: 86.4–95.0%], the specificity was 91.5% [95% CI: 86.8–95.0%], and the area under the receiver operating characteristic curve (AUC) was 0.92 [95% CI: 0.88–0.94] (*p* < 0.0001). The ROC curve is illustrated in Figure 1. Among the 387 tested data, 354 cases provided no difference between the operators’ visual assessments (ground truth) and the automatically computed results provided by the application (Figure 2), leading to an accuracy of 91.5% [95% CI: 88.2–94.1%] (Table 2). The MCC was 0.83, which is a factor of a very good prediction.

**Figure 3:**
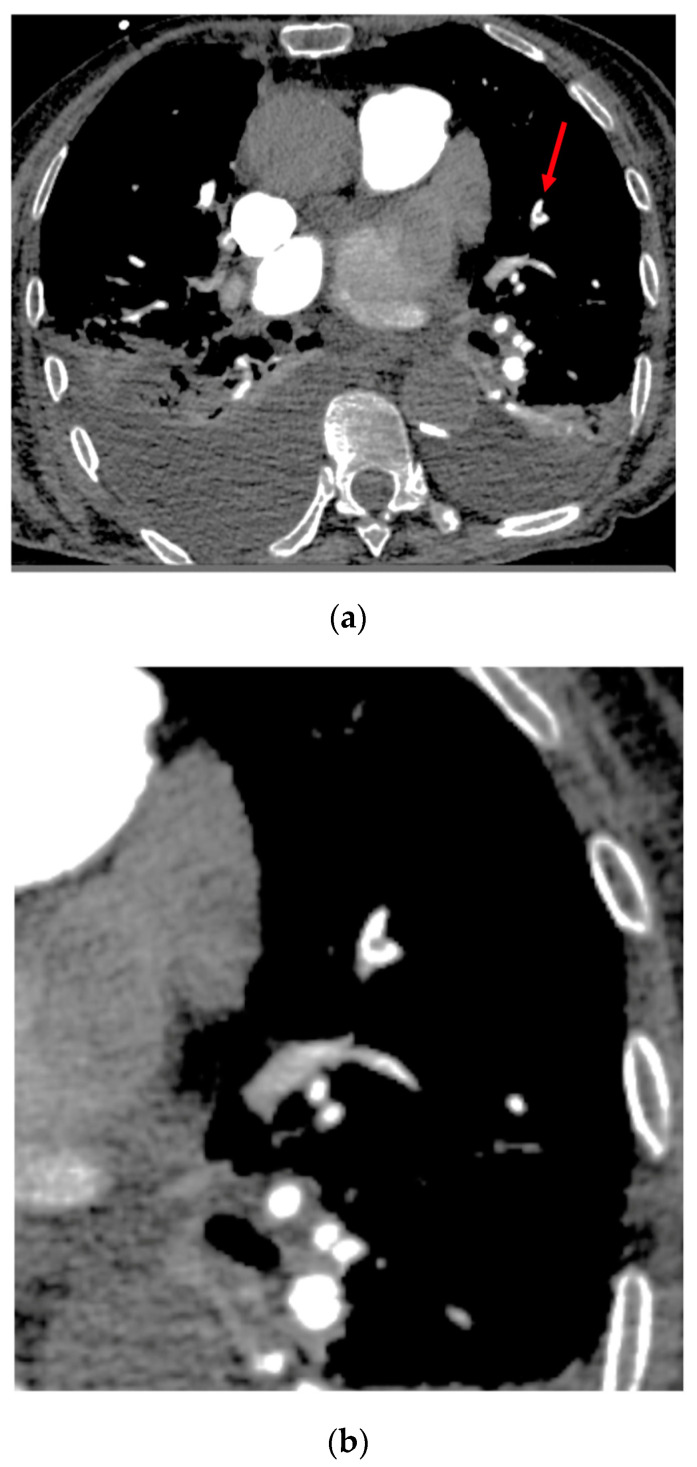
Case of the false negative response of the algorithm in the left upper lobe. Top (**a**): Axial image showing excellent opacification of pulmonary arteries. The red arrow shows the small filling defect within the termination of the segmental artery just close to the origin of subsegmental arteries. Note the presence of bilateral pleural effusion. Bottom (**b**): Axial image targeted on the left lung illustrating the presence of PE.

There were 16 FNs with a miss rate for PE of 8.6% (16/186 positive cases). Among them, six corresponded to small chronic PEs. The location and cause of the other FN cases are reported in Supplementary Materials—Table S2. Among these 16 FN cases, three were a cause of disagreement between the radiologists in charge to provide the ground truth, with huge difficulty to visualize the clots on the images. Several missed PEs were at the limit of subsegmental arteries and close to the partial volume effect artifacts (Figure 3).

**Figure 4:**
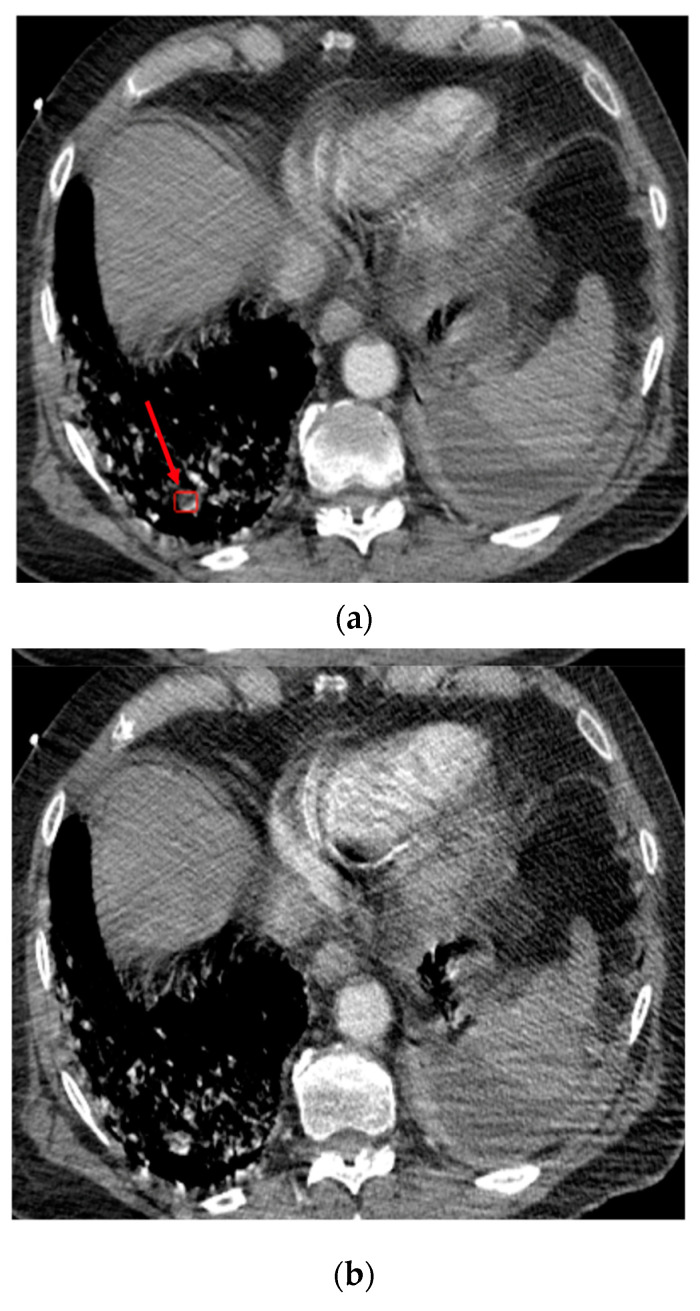
Case of the false positive response of the algorithm in the right lower lobe. Top (**a**): Axial image at the level of lung bases. The red arrow shows the pseudo-lesion. Bottom (**b**): Subsequent axial image showing motion artifacts blurring the pseudo-lesion.

A total of 17/201 (8.5%) cases were false positives. Among them, six cases were complicated cases and were the subject of disagreements between the experts. Six PEs were correctly detected within subsegmental arteries, but these were the cases considered as “negative” by the readers, since the application was not designed/trained to flag subsegmental arteries. The other false positives were due to the presence of important noise, streak and/or motion artifacts (Figure 4), or the partial volume effect artifact. The location and cause of the false positives are reported in Supplementary Materials—Table S3.

### 3.4. Positive and Negative Predictive Values

Positive and negative predictive values with varying prevalence values (from 10 to 50%, increment of 5%) are provided in Table 3. The negative predictive values exceeded 91% and varied from 91.4% for a PE prevalence of 50% to 99.0% for a prevalence of 10%. The positive predictive values ranged between 54.6% for a prevalence of 10% and 91.5% for a prevalence of 50%, and exceeded 82% for a prevalence higher than 30%.

### 3.5. Stratified Analysis

The stratifications are provided for the subgroups of imaging acquisitions and scan parameters (scanner makers, number of detector rows and slice thickness), and the patient groups (U.S. regions, patient age and sex). The algorithm performance across all subgroups is presented in Table S1 (Supplementary Materials). The stratified statistical analysis showed sensitivities between 86.7% and 100%, and specificities between 82.4% and 95.5%. Therefore, for all categories, and for each group, the sensitivities and specificities were higher than 82%.

### 3.6. Time-to-Notification

The application was run with the following hardware specifications: CPU: 8 threads at 3.0+ GHz and RAM: 16 GB. Time-to-notification (TTN) was calculated for all 387 cases. The mean TTN ± SD was 43.6 ± 9.9 s with 42.6 and 44.6 s for the lower and upper 95% CI limits, respectively. The median value was 41.7 s. The minimum and maximum TTN were 26.2 and 82.0 s, respectively.

## 4. Discussion

We conducted a retrospective, multicenter, and blinded study to validate the standalone performance of a new AI-powered application for suspected PE triage and prioritization on chest CTA images and to test its generalizability. The data came from multiple U.S. clinical sites acquired on five different scanner makers and 41 different scanner models. They included 186 (48.1%) positive PE cases. This validation study focused on assessing the standalone diagnostic performance of the algorithm when compared to the ground truth established by three U.S. board certified expert radiologists. The results showed an accuracy of 91.5% when compared to the visual assessment of the operators. The sensitivity and specificity exceeded 91% with a ROC-AUC of 0.92. In addition, the TTN ranging between 26 and 82 s was well-adapted for practical use in emergency radiology.

According to the literature, other DL solutions achieved performances inferior or similar to those of CINA-PE, but with important limitations. Indeed, Ajmera et al. developed a 2D segmentation model consisting of U-Net architecture to detect PEs on CTAs. They tested the model performance on a single external dataset consisting of CTAs from 251 patients of whom 55 presented PE. Sensitivity and specificity were 80% and 74% respectively with an accuracy of 0.76 [25]. Huang et al. developed and evaluated an end-to-end DL model capable of detecting PE using the entire volumetric CTA imaging examination. This model achieved an AUC of 0.84 for automatic PE detection on the hold out test set and 0.85 AUC on the external datasets [20]. Liu et al. trained a DL model on the CTAs of 590 patients (460 with PE and 130 without PE) for the segmentation of the clots. Validation was performed on an in-house dataset made of 288 patients (186 with PE and 102 without PE). The AUC was 0.92, the sensitivity was 94.6%, but the specificity was only 76.5% [22]. Huhtanen et al. also developed a DL model trained and validated on 600 CTAs, and tested their model on 200 CTAs. The model achieved good performances (AUC 0.94, sensitivity 93.5% and specificity 86.6%). However, they only used data from a unique institution and the majority of the data was acquired with CT scanners from one vendor [24]. Finally, Weiker et al. trained and validated a DL solution comprising a Resnet architecture (Briefcase, Aidoc Medical) on 28,000 CTAs acquired at various institutions for automatic PE prioritization and triage. The diagnostic accuracy, measured on a test dataset including 232 positive and 1233 negative examinations was high with a sensitivity of 92.7% and a specificity of 95.5% [23]. However, the dataset selection for the test was made from a single institution, missing the evaluation of potential generalizability. On the other hand, in a more recent publication, investigators used the Briefcase software to retrospectively assess the presence or absence of PE on CTA examinations performed in various emergency departments and sent to interpretation centers of three French cities (Bordeaux, Lyon, and Marseille), thus reinforcing the validation of the algorithm [27].

The objective of CINA-PE is to detect acute PE on CTAs. However, even if chronic PE is not generally an urgent finding that warrants immediate triage or an immediate need to treat the patient, particular attention must be paid for “chronic PEs”, which may be missed by the application as in six cases of our series, especially in the presence of acquisition artifacts. The other FNs observed in our study were difficult cases with PE locations close to the partial volume effect artifacts, at the limit of subsegmental arteries, with bad quality of the image acquisitions, or in the case of complete occlusion of the artery.

As the CINA-PE application was not trained to flag subsegmental arteries, the cases of subsegmental PEs detected by the algorithm and confirmed by the radiologists were considered as false positives. Indeed, as reported by Carrier et al., the subsegmental pulmonary embolism diagnoses identified by CTA are unlikely to be clinically relevant and may not be worth diagnosing, provided that there is no evidence of deep vein thrombosis [28]. The other FP cases also reported in the literature [23,24] were due to the partial volume effect with hilar lymph nodes, pulmonary veins, or streaks of beam-hardening artifacts. Consequently, any positive result detected by the algorithm leads to an automatic notification for the radiologist who has to determine the quality of images and to decide if the case is positive, negative, or indeterminate with an option of additional investigations.

In clinical practice, the majority of CTAs performed for suspicion of PE are negative [29,30,31]. According to the literature, the yield of positive cases varies from less than 10 to 20–30% [29,31,32,33]. As prevalence has a strong influence on positive and negative predictive values, this might translate into an application site-dependent performance. When the prevalence values varied from 10% to 30% in our study data, the extrapolated positive predictive value varied from 54.6% to 82.2% while the negative predictive value moved from 99% to 96.1%. In all cases of positive results detected by the algorithm, the radiologist was informed by an automatic notification, then had to review the images flagged by the software and decide between TP and FP. For the cases not flagged by the algorithm, they were ultimately reviewed through the standard of care workflow without interruption.

The main clinical usefulness of the proposed DL-based solution is currently the prioritization of CTAs detected as positive for PE for an urgent and prompt interpretation by the radiologist. This is particularly beneficial when the workload increases in emerging settings. Beyond this primary objective, such automated triage and prioritization application could potentially improve the radiologist’ performance and ultimately result in better patient clinical outcomes. However, the potential benefit of using this application to improve the performance of radiologists in diagnosing PE on CTAs remains to be assessed. Hence, our next objective step will be to compare the diagnostic performances of the AI-based device alone and radiologists alone with those of radiologists with full access to the AI output during their practice.

Our study presented some limitations. First of all, a direct comparison between the DL algorithm and the performance of the radiologists was not carried out. Second, we did not use a multitask DL approach like that of other investigators who developed a two-phase multitask learning method that could recognize the presence of PE and its properties as the position, whether acute or chronic, and the corresponding right-to-left ventricle ratio, thereby reducing FN diagnoses [34]. Furthermore, we did not integrate both clinical and imaging data as Huang et al. did, who developed different multimodal fusion model architectures that were capable of utilizing both pixel data from CTAs and clinical patient data from the electronic health record to automatically classify PE cases [35].

Key Points:

1. To help radiologists to prioritize exams with a high suspicion of pulmonary embolism (PE), a deep learning (DL) algorithm was trained on 4795 CT angiograms from multiple U.S. clinical centers and adequately distributed in terms of vendors, patient age, slice thickness, and kVp.
2. In a retrospective, multicenter, and blinded standalone validation study, the DL algorithm achieved an area under the receiver operating characteristic curve (AUC) of 0.92 [95% CI: 0.88–0.94] (*p* < 0.0001).
3. Both the training and validation of the algorithm on data from multiple clinical sites through the U.S. and acquired on multiple scanner models promotes the generalizability of its use.

## 5. Conclusions

The CINA-PE AI-powered application developed to prioritize suspected PE on CTAs for immediate reading by radiologists was trained and validated on multicentric real-world data adequately distributed in terms of vendors, CT scanner models, patient age and sex, slice thickness, and KVP. The validation phase of CINA-PE achieved a high accuracy (0.92 AUC) to detect suspected acute PEs on CTAs. The great heterogeneity of datasets used for both training and validation of the DL-based software should guarantee enough robustness for generalization of its use. The potential clinical benefit of using such an automatic PE detection system to improve the performance of the radiologists, and ultimately result in better patient clinical outcomes, remains to be assessed.

## Figures and Tables

**Table 1:** The inclusion criteria were chest CTAs acquired on adults (≥18 years old) that met the following acquisition requirements.

Acquisition Parameters
Matrix size ≥ 512 × 512 (rectangular matrix accepted)
Axial acquisition only
Slice thickness ≤ 2.5 mm with no gap between successive slices
Radiation dose parameters: 60 kVp to 160 kVp
Reconstruction diameter above 200 mm
Contrast intensity: Minimum opacification the in the main pulmonary artery (mPA) = 180 HU (Hounsfield units)
Contrast timing: Minimum opacification ratio between the mPA and the ascending aorta = 0.95
Soft tissue reconstruction kernel

**Table 2:** Statistical findings for CINA-PE.

Statistical Findings			
TP	170	Sensitivity [95% CI]	91.4% [86.4–95.0%]
FN	16	Specificity [95% CI]	91.5% [86.8–95.0%]
TN	184	Accuracy [95% CI]	91.5% [88.2–94.1%]
FP	17	AUC [95% CI]	0.92 * [0.88–0.94]

* Significance level *p* (area = 0.5) < 0.0001.

**Table 3:** Summary of positive and negative predictive values for CINA–PE application, completed by varying prevalence values (from 10% to 50%, increments of 5%).

Prevalence (%)	CINA—PE Positive Predictive Value (%)	CINA—PE Negative Predictive Value (%)
10	54.6	99.0
15	65.6	98.4
20	73.0	97.7
25	78.3	97.0
30	82.2	96.1
35	85.3	95.2
40	87.8	94.1
45	89.8	92.9
50	91.5	91.4

## Data Availability

Not applicable.

## Supplementary Materials

The following supporting information can be downloaded at: https://www.mdpi.com/article/10.3390/diagnostics13071324/s1.

## Abbreviations

PE = pulmonary embolism; CTA = computed tomography angiogram; DL = deep learning; CNN = convolutional neural network; TP = true positive; FP = false positive; TN = true negative; FN = false negative; AUC = area under the receiver operating characteristic curve; MCC = Matthews correlation coefficient.
