# Supplementary material for: Deep Learning-Based Algorithm for Automatic Detection of Pulmonary Embolism in Chest CT Angiograms
Source: Diagnostics (Basel). 2023 Apr 3;13(7):1324. doi: 10.3390/diagnostics13071324 (PMC10093638; doi:10.3390/diagnostics13071324)
Supplement: Supplementary file 1 [file diagnostics-13-01324-s001.zip › diagnostics-2241156-supplementary.pdf]

Supplementary Material

Table S1: Algorithm performances across all subgroups of imaging acquisitions and scan parameters, and patients' groups (US regions, patients' age and sex).

PARAMETER	CATEGORY	TP	FN	TN	FP	Sensitivity [95% CI]	Specificity [95% CI]
TYPE	All	170	16	184	17	91.4% [86.4% – 95.0%]	91.5% [86.8% – 95.0%]
GENDER	Male	85	106	8	6	93.41% [86.2% - 97.5%]	92.98% [86.6% - 96.9%]
	Female	85	78	9	10	89.47% [81.5% - 94.8%]	89.66% [81.3% - 95.2%]
AGE	18 ≤ Age < 40	23	0	14	3	100% [85.2% - 100%]	82.35% [56.6% - 96.2%]
	40 ≤ Age ≤ 60	61	3	45	4	95.31% [86.9% - 99.0%]	91.84% [80.4% - 97.7%]
	Age > 60	86	13	125	10	86.87% [78.6% - 92.8%]	92.59% [86.8% - 96.4%]
SLICE THICKNESS	ST < 1.5 mm	75	6	71	8	92.59% [84.6% - 97.2%]	89.87% [81.0% - 95.5%]
	1.5 mm ≤ ST ≤ 2.5 mm	95	10	113	9	90.48% [83.2% - 95.3%]	92.62% [86.5% - 96.6%]
DETECTOR ROWS	4 < NDR ≤ 8	0	0	0	0	NA	NA
	8 < NDR ≤ 16	24	0	14	1	100% [85.8% - 100%]	93.33% [68.1% - 99.8%]
	16 < NDR ≤ 32	22	3	31	2	88% [68.8% - 97.5%]	93.94% [79.8% - 99.3%]
	32 < NDR ≤ 64	94	10	105	12	90.38% [83.0% - 95.3%]	89.74% [82.8% - 94.6%]
	64 < NDR ≤ 128	19	2	21	1	90.48% [69.6% - 98.8%]	95.45% [77.2% - 99.9%]
	128 < NDR ≤ 256	0	0	0	0	NA	NA
	256 < NDR ≤ 320	1	0	0	0	100% [2.5% - 100%]	NA
US REGIONS	Continental	23	3	25	2	88.46% [69.9% - 97.6%]	92.59% [75.7% - 99.1%]
	Northeast	83	6	93	5	93.26% [85.9% - 97.5%]	94.9% [88.5% - 98.3%]
	Pacific	16	1	26	3	94.12% [71.3% - 99.9%]	89.66% [72.7% - 97.8%]
	Southeast	48	6	40	7	88.89% [77.4% - 95.8%]	85.11% [71.7% - 93.8%]

**MANUFACTURER**	**GE MEDICAL SYSTEMS**	105	12	100	10	89.74% [82.8% - 94.6%]	90.91% [83.9% - 95.6%]
	**PHILIPS**	15	0	15	2	100% [78.2% - 100%]	88.24% [63.6% - 98.5%]
	**SIEMENS**	36	2	51	4	94.74% [82.3% - 99.4%]	92.73% [82.4% - 98.0%]
	**TOSHIBA/ CANON**	13	2	18	1	86.67% [59.5% - 98.3%]	94.74% [74.0% - 99.9%]
	**PNMS**	1	0	0	0	100% [2.5% - 100%]	NA

Table S2: CINA–PE standalone validation study: Details regarding the found false negative (FN) cases.

CASE N°	False negatives' descriptions
FN #1	Missed right very small segmental **Chronic PE**<br>**This case was subject of consensus**<br>**Presence of vena cava artifacts**<br>Presence bronchiolitis
FN #2	Missed right lobar and segmental **Chronic PE**<br>Presence of **linear filling defect** within the PE (more peripheral filling defect)<br>Presence of ground glass opacity<br>Presence of pleural fluid
FN #3	Missed right very small segmental PE<br>Presence of tots of lung nodules
FN #4	Missed left small segmental PE located close to a **partial volume effect artifact**<br>The location of this PE is **limit subsegmental**<br>Presence of bilateral pleural effusions
FN #5	Missed right very small segmental **Chronic PE**<br>Presence of **acquisition artifacts**<br>Presence of atelectasis
FN #6	Missed very small left lobar and segmental **Chronic** PE close to **partial volume effect artifacts**<br>**This case was subject of consensus**<br>Presence of multiple noncalcified pulmonary nodules<br>Presence of calcified mediastinal/subcarinal lymph node
FN #7	Missed right interlobar **Chronic PE**<br>Presence of **linear filling defect** within the PE<br>**This case was subject of consensus**
FN #8	Missed left segmental PE<br>Presence of **bad contrast filling**

FN #9	Missed right main **Chronic PE**<br>PE located inside of **important motion artifacts** and **cava vena artifacts**
FN #10	Missed right segmental PE in presence of **volume partial effect artifacts**<br>Presence of **bad contrast filling**
FN #11	Missed left small segmental PE<br>**Nosy images**<br>**Presence of bad contrast filling**<br>Presence of pulmonary edema, pneumonia, and pleural effusion
FN #12	Missed right segmental PE<br>The location of this PE is **limit subsegmental**<br>**Complete occlusion of the artery**
FN #13	Missed bilateral small segmental PE<br>The location of this PE is **limit subsegmental**
FN #14	Missed right segmental PE close to **hilar lymph node** and **partial volume effect artefacts**<br>Presence of bunch of nodules, pleural effusion, and ground glass opacity<br>Presence of metastasis
FN #15	Missed left main PE in presence of **tumor near to the occlusion**<br>Presence of metastatic nodules<br>Presence of Pneumonia and complex pleural effusion
FN #16	Missed right main PE **at the limits of chronic PE transformation**<br>Presence of an important pneumothorax in the right lung.

Table S3: CINA–PE standalone validation study: Details regarding the found false positive (FP) cases.

CASE N°	False positives' descriptions
FP #1	PE wrongly detected within **a pulmonary vein**<br>**Important vena cava artifacts**<br>**Contrast mixing**
FP #2	Large **subsegmental** PE **correctly** detected<br>**This case was subject of consensus**<br>Presence of atelectasis, consolidation, and pleural effusion
FP #3	**Very bad quality images**: PE wrongly detected in **the pleural effusion** in presence of **very noisy images** and **important streak artifacts**<br>Presence of atelectasis, pneumothorax, and pleural effusion
FP #4	PE wrongly detected **in the vena cava artifacts**.<br>**This case was subject of consensus**<br>Presence of nodule, atelectasis, and pneumonia<br>Presence of pneumothorax and pleural effusion<br>Presence of drainage catheter
FP #5	PE wrongly detected in the **hilar lymph node**<br>Presence of emphysema, consolidation, and atelectasis<br>Presence of pneumothorax and pleural effusion
FP #6	PE wrongly detected in the **vena cava artifacts**.<br>**Poor contrast filling and anatomic distortion**<br>Presence of ground glass opacity and lung infiltration<br>Presence of pneumothorax, and atelectasis<br>Presence of emphysema
FP #7	Small PE **correctly** detected within a **subsegmental** artery<br>Presence of atelectasis
FP #8	PE wrongly detected in the **cava vein artefacts**.<br>Presence of emphysema<br>Presence of nodule
FP #9	PE wrongly detected within a **saccular aneurysm** stuck to the main pulmonary artery.<br>Presence of **central venous catheter** with the tip in the brachiocephalic vein<br>Presence of atelectasis/consolidation, and pleural effusion
FP #10	PE **correctly** detected within a **subsegmental** artery<br>**This case was subject of consensus**<br>**Contrast mixing**<br>Presence of **streak artifacts and important motion artifacts**<br>There is an aortic repair (graft)<br>An endotracheal tube is in place<br>Presence of atelectasis and lung infiltration

FP #11	PE **correctly** detected within **subsegmental** artery with **partial volume effect artifacts**<br>Presence of **bad contrast filling**<br>**Noisy images**<br>Presence of atelectasis, and nodules
FP #12	**Partial volume effect artifacts** (crossing between a vein and an artery)<br>Presence of **bad contrast filling, and motion artifacts**<br>Presence of calcified pleural plaques
FP #13	Streak artifact (graft)<br>Presence of **streak artifacts, bad contrast filling, and motion artifacts**<br>**This case was subject of consensus**
FP #14	PE correctly detected in a **subsegmental** artery<br>**This case was subject of consensus**<br>Presence of pulmonary nodules
FP #15	PE correctly detected in a **subsegmental** artery, although very close to the segmental artery<br>**This case was subject of consensus**
FP # 16	PE within **a pulmonary vein**<br>Presence of ground glass opacity
FP #17	PE within **vena cava artifacts**
